# The Part and the Whole

**Published:** 1917-05-19

**Authors:** 


### THE PART AND THE WHOLE.

By CADUCEUS.

A real sage of our day—he never speaks in public—has brought new light by his contrast of two kinds of mind: the "classical" one, which thinks the whole greater than the part, and the "decadent," which thinks just the reverse. The opposition is stated with careless boldness, so that the "classicists" seem to have the best of it. But in practice this may not be so, in the future, at least, or even now. If, say, journeying to the suburbs in a motor-'bus, one turned to one's neighbour and asked him freely what sort of a job he thought a Cabinet Minister's was, the probability is no answer would be forthcoming, truisms on such occasions being unattractive, and the questioned person being entirely convinced that of all human stations this was the highest and the most onerous. Or, if one dared to inquire of the good lady opposite why she carried on her knee those two "best sellers," her also lacking reply could be supplied quite safely, to the effect, namely, that she read Mr. Garvice and Mrs. Barclay because of the interest, the agreeable emotions, inspired by their stories' subjects, or rather subject; and, indeed, the shade of Matthew Arnold, hearing such an answer, would smile with true approval. But it would not be safe to expect much admiration of prominent politicians in the Chelsea Art Club or a big laboratory, or from an unvenal journalist. These men (the criticism now would be) are mostly by nature rumbustious fellows, fond of the sound of their own voices, knowing thoroughly the commoner human motives, who have worked hard as lawyers, and, following the thinkers of the age at a respectful distance and public opinion very closely, settle these "great national questions" in committee precisely as disputes and bargains and contracts are settled every day in every quarter of every town in the country. Their intelligences are a little above sample of their type, but that type is a widely distributed one, of no lofty order at all. And there, of course, are plenty of superior persons to point out that Flaubert's scanty output, in years of labour, is frequently inferior to a book for boys in the way of incident, much of the difference consisting in the style of the narration. Again, if to a "classical" man and a "decadent" one, one were to mention the position of the Presidents of France and America in the last Coronation procession, riding in plain vehicles a long way behind Continental princelings, then one of the two would consider the riches of the United States, and the wit and art of Paris, compared with the productions of a castle or two and a few square kilometres of forest, a topic much more relevant to the discussion than the other would. The one to enlarge upon this suggestive theme would be the "decadent."

This special and peculiar "decadence" (the term should be conceived etymologically; its use is as uncompromising, as empty of concessions to the plain man as the phrasing of the distinction itself) has so far been traced in somewhat aristocratic associations. But it is to be noticed in quarters which are anything but that. Before the war came to interrupt thought or action in almost all other directions, there was the movement known differently as syndicalism, or guild socialism, which, though based most of all on economic grounds, yet surely owed not a little to this feeling of antagonism that the specialist has for the non-specialist. These last words bring us, perhaps not over-soon, to the medical interest of our subject. The medical deputation which suggested a separate Ministry of Health put forward a "decadent" proposal, and Mr. Walter Long answered in characteristically "classic" fashion when he deprecated any fission of the Local Government Board. But the conflict may be an internecine one. The medical officer of health, who wants to be the chief venereal officer of his district, as he is already the tuberculosis and school medical officer, the nominal director of the laboratory, the baby centre adviser, and the supervisor of dental, obstetric and ophthalmological agencies, and who opposes any "hiving off" of any of these departments from his office, such as in the past River Boards did, is just as "classical" as the politician; the future has changes in store for both of them. M. Rolland claims that in France government and administration have for some time past attracted inferior men. Probably this is the effect of the complexity of modern civilised life, affording openings for superior talent in its details, its tributaries, not in the sluggish main current which descends from antiquity. And as life becomes ever more heterogeneous, so this phenomenon is likely to become increasingly evident. Some alteration of relative status will have to occur: the depositary of elementary authority must go lower and lower, and his highly cultured subordinate rise higher and higher. Such a process could not fail to be, on the whole, beneficial to the medical profession.

[*Our correspondent appears to assume that the organisation of a Ministry of Health would turn every medical officer of health into a Controller of the activities of the local profession. We do not admit the validity of such an assumption, but were it justified, our advocacy of a Ministry of Health would be sensibly modified.*—Ed. The Hospital.]

